# Correlation between the Charlson comorbidity index and skeletal muscle mass/physical performance in hospitalized older people potentially suffering from sarcopenia

**DOI:** 10.1186/s12877-019-1395-5

**Published:** 2019-12-23

**Authors:** Ge Gong, Wenhui Wan, Xinghu Zhang, Yu Liu, Xinhui Liu, Jian Yin

**Affiliations:** 1 Department of Geriatrics, Jinling Hospital, Medical School of Nanjing University, No.305, Zhongshan east road, Nanjing, 210002 China; 2 Department of Orthopedics, The Affiliated Jiangning Hospital with Nanjing Medical University, Nanjing, 211100 China

**Keywords:** Charlson comorbidity index, Sarcopenia, Skeletal muscle mass, Physical performance, Hospitalized older people

## Abstract

**Background:**

Sarcopenia is a decrease in skeletal muscle mass, physical performance, and muscle strength in older people. In this study, we aimed to explore the correlation between comorbidity and skeletal muscle mass and physical performance in older people.

**Methods:**

This retrospective study included 168 subjects. Their medical history, physical function, computed tomography (CT) chest scans, and blood tests for nutrition were evaluated. The patients were divided into two groups: (1) a low muscle mass group and (2) a normal muscle mass group. Multivariate analysis of variance was used to compare multiple sets of mean vectors.

**Results:**

Overall, 72.02% of the subjects had a low skeletal muscle index (SMI) and low gait speed. The patients with low skeletal muscle mass and physical performance were older, had more serious comorbidities, and had longer average hospitalization periods and lower albumin and hemoglobin levels. Subjects with a high Charlson comorbidity index (CCI) were more likely to be in the sarcopenic group than in the non-sarcopenic group. In addition, there was a linear correlation between the CCI and SMI (r = − 0.549, *P* < 0.05), and between the CCI and gait speed (r = − 0.614, *P* < 0.05). The area under the curve (AUC) value for low skeletal muscle mass with the CCI was 0.879.

**Conclusions:**

We identified an independent association between comorbidity and skeletal muscle mass/physical performance by researching the correlation between the CCI and SMI/gait speed. Our results suggested that the CCI score may have important clinical diagnostic value for sarcopenia.

## Background

With the gradual aging of the population, sarcopenia has attracted increased attention among geriatricians. In 2010, a definition for sarcopenia was published by the European Working Group on Sarcopenia in Older People (EWGSOP). They defined it as a syndrome characterized by progressive and generalized loss of skeletal muscle mass and strength with a risk of adverse outcomes such as physical disability, poor quality of life, and death [1]. In 2018, the working group met again (EWGSOP2) to update the definition of sarcopenia [2]. In this definition, EWGSOP2 reported that sarcopenia is probable when low muscle strength is detected and sarcopenia diagnosis is confirmed by the presence of low muscle quantity or quality. Sarcopenia is deemed to be severe in cases of low muscle strength, low muscle quantity/quality, and low physical performance. It has a relatively high incidence among the elderly [3]. Studies have shown [4] that the incidence of sarcopenia is 5–13% among those 60–70 years of age and 11–50% among those over 80 years of age. Sarcopenia is a progressive and generalized skeletal muscle disorder that is associated with increased likelihood of adverse outcomes including falls, fractures, physical disability, and mortality [2]. Consequently, the occurrence of sarcopenia leads to a decline in the quality of life, frailty, and even death among older people [5, 6].

However, older people often have several chronic diseases. The incidence of comorbidity and the coexistence of two or more diseases [7] increases with age in older people. The morbidity and mortality of older people with comorbidities are high, and their quality of life is low. Many types of disease are found in older people with comorbidities. For example, chronic obstructive pulmonary disease (COPD) [8], stroke [9], Parkinson’s disease [10], and chronic kidney disease [11] are likely to co-occur with problems such as weakness, malnutrition, and disability. When two or more chronic diseases coexist, the risk of falling and disability in older people is much higher than in those without comorbidity [12, 13].

The Charlson comorbidity index (CCI) [14] is currently the most commonly used comorbidity assessment tool. Some studies have been conducted on the association between comorbidity and sarcopenia [15–17]. However, whether the CCI can be used to assess the risk of sarcopenia is unclear. In this study, we reviewed the comorbidity characteristics of chronic diseases in older people potentially suffering from sarcopenia and studied the association between skeletal muscle mass/physical performance and CCI scores.

## Methods

### Participants and study design

**Fig. 1:**
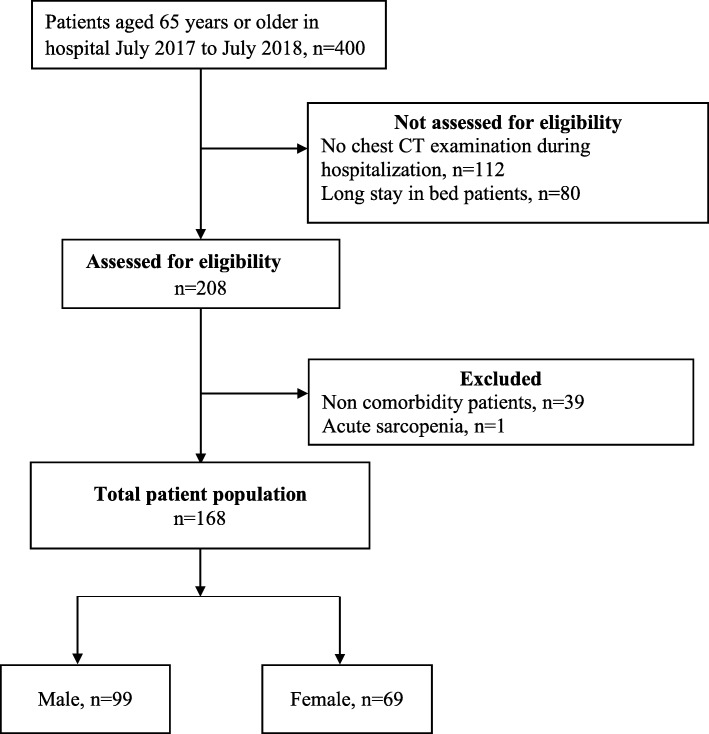
Study flowchart. Patients who met the inclusion and exclusion criteria for the study population. CT: computed tomography

A retrospective analysis was conducted on older people, 65-years-old or older, who were admitted to the Department of Geriatrics, Jinling Hospital, Medical School of Nanjing University between July 2017 and July 2018. Data were collected from the hospital’s electronic medical record system and this study was approved by the Ethical Committee of Jinling Hospital. The exclusion criteria were as follows: (1) chest CT examination not performed during hospitalization; (2) long time (> 1 year) spent in bed; (3) height and weight measurements not taken at admission; (4) patients without comorbidity, and (5) patients with acute sarcopenia—e.g., caused by steroid treatment, neurodegenerative diseases, hip or knee replacement in the previous six months, stroke in the previous six months, or deep vein thrombosis in the previous six months. Only patients who had undergone assessments related to this study were included and patients were enrolled consecutively (Fig. 1).

For each patient, the following data were collected: age, sex, smoking status, body mass index (BMI), comorbidity index, length of stay, hospital mortality, chest CT, and laboratory indicators (albumin and hemoglobin).

### Sarcopenia

According to the test for sarcopenia [18], as developed by the Asian Working Group for Sarcopenia (AWGS), physical performance is generally evaluated on the basis of the individual’s gait speed and the established cut-off value for normal gait speed (< 0.8 m/s, 6 m course). Performance was measured with a stopwatch by one nurse.

**Fig. 2:**
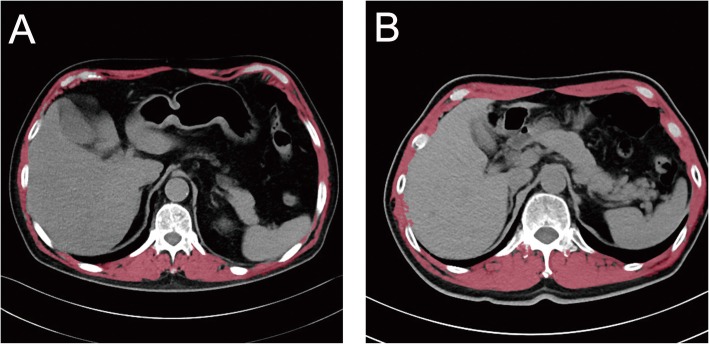
Thoracic SMI with CT of low skeletal muscle mass and normal skeletal muscle mass. Axial computed tomography images of the twelfth thoracic region, with skeletal muscle highlighted in red (40 to 80 Hounsfield units). (**a**) Example of an elderly male patient with a low T12 SMI (< 42.6 cm^2^/m^2^). (**b**) Example of an elderly male patient with a high T12 SMI (> 42.6 cm^2^/m^2^). SMI: skeletal muscle index, CT: computed tomography

In addition, chest CT scans were examined and the diagnosis for sarcopenia was based on an internationally recognized skeletal muscle index (SMI) and sex-specific definition. In order to save extra cost and injury, we chose SMI values derived from the total muscle area (in cm^2^) divided by the square of the height (in m) [19, 20] at the twelfth thoracic vertebra (T12) level. The diagnosis of sarcopenia was as proposed by Nemec et al. [19] of Harvard Medical School: SMI values less than 42.6 cm^2^/m^2^ for men and 30.6 cm^2^/m^2^ for women. All data were measured independently by two physicians with CT reading expertise. In this study, all patients underwent chest CT scans without contrast agent. PACS software (SITS 3.6, Philips Healthcare, Guildford, UK) was used to measure the pedicle muscle area (cm^2^) in the chest CT (Fig. 2a and b).

### Charlson comorbidity index (CCI)

The CCI is an index used to predict the risks of short-term and long-term patient mortality. It consists of three parts: disease assessment, severity assessment, and scoring. It includes 16 diseases: myocardial infarction, score 1; heart failure, score 1; peripheral arterial obstructive disease, score 1; cerebrovascular disease, score 1; senile dementia, score 1; COPD, score 1; connective tissue disease, score 1; gastroduodenal ulcer, score 1; diabetes, score 1 (involvement of other organs, score 2); chronic kidney disease, score 2; hemiplegia, score 2; leukemia, score 2; malignant lymphoma, score 2; tumor, score 2 (metastasis, score 6); liver disease, score 1 (moderate to severe, score 3); and AIDS, score 6. Age-based scoring starts at the age of 50 years, with a 1 point increase for every 10 years above age 50. The CCI has been used extensively in the medical literature for comorbidity assessment [21].

### Statistical analysis

Measurements taken by the two researchers were averaged for the purposes of outcome analyses. Statistical analyses were performed in SPSS 20.0 (IBM SPSS Statistics for Windows, Armonk, NY, USA). In addition to descriptive statistics, the mean and standard deviation were determined for numeric variables, and the number and percentage were determined for categorical variables. Multivariate analysis of variance was used for comparison of multiple sets of mean vectors. Pearson’s and Spearman’s correlation analysis and linear regression analysis were used for correlation analysis, and *P* < 0.05 was considered statistically significant. Meanwhile, PASS 15.0 (Power Analysis and Sample Size) software was used to calculate the statistical power.

## Results

### Participant characteristics

Of the 400 patients who were > 65 years old, 232 were excluded (Fig. 1). Consequently, the final study population consisted of 168 patients, of whom 99 (58.93%) were men and 69 (41.07%) were women. Sample size estimation and power analysis revealed a sample size of 168, with α = 0.05 (both sides), achieved 100% power. The mean age was 83.34 ± 7.32 years. The mean duration of stay was 14.19 ± 11.07 days, and the mean BMI was 24.52 ± 3.19 kg/m^2^. In addition, the mean gait speed was 0.69 ± 0.25 m/s. Eighteen (10.71%) patients were smokers. There was no significant difference in age, duration of stay, gait speed, or BMI between men and women (*P* > 0.05). Detailed demographics and clinical data are presented in Table 1. Among the patients, the incidence of cerebrovascular disease was highest (58.33%), followed by diabetes (38.69%). In addition, 54 (32.14%) patients scored 6 points on the CCI. These measurements are shown in Table 2.

**Table 1:** Characteristics of Patients

Characteristic	All(*n* = 168)	Men(*n* = 99)	Women(*n* = 69)	*P*-value *(Men* vs. *Women)*
Age(y)	83.34 ± 7.32	84.15 ± 7.54	82.17 ± 6.88	0.085
Duration of stay(d)	14.19 ± 11.07	15.51 ± 13.28	12.30 ± 6.38	0.065
BMI (kg/m^2^)	24.52 ± 3.19	24.37 ± 3.40	24.75 ± 2.86	0.44
Gait speed(m/s)	0.69 ± 0.25	0.67 ± 0.26	0.71 ± 0.25	0.301
Smoking, n (%)	18 (10.71)	15 (15.15)	3 (4.35)	0.026

Age, Duration of stay and *BMI* (mean ± SD)

*P-value for difference between subgroups with independent sample t test

**Table 2:** Comorbidity disease and CCI score

	All(*n* = 168)	Men(*n* = 99)	Women(*n* = 69)
Comorbidity disease, n (%)			
MI	22 (13.10)	13 (13.13)	9 (13.04)
CHD	20 (11.90)	17 (17.17)	3 (4.35)
PAOD	6 (3.57)	3 (3.03)	3 (4.35)
CVD	98 (58.33)	68 (68.69)	30 (43.48)
SD	11 (6.55)	2 (2.02)	9 (13.04)
COPD	20 (11.90)	11 (11.11)	9 (13.04)
CTD	6 (3.57)	0 (0)	6 (8.70)
GU	49 (29.17)	31 (31.31)	18 (26.09)
DM	65 (38.69)	50 (50.51)	15 (21.74)
CKD	22 (13.10)	13 (13.13)	9 (13.04)
HP	2 (1.19)	2 (2.02)	0 (0)
CL	0 (0)	0 (0)	0 (0)
ML	1 (0.60)	1 (1.01)	0 (0)
tumor	49 (29.17)	28 (28.28)	21 (30.43)
LD	14 (8.33)	11 (11.11)	3 (4.35)
CCI score, n (%)			
4	11 (6.55)	4	7
5	33 (19.64)	7	26
6	54 (32.14)	33	21
7	24 (14.29)	21	3
8	22 (13.10)	22	0
9	7 (4.17)	4	3
10	3 (1.79)	3	0
11	5 (2.98)	2	3
12	8 (4.76)	2	6
13	1 (0.60)	1	0

*MI* Miocardial infarction, *CHD* Chronic heart failure, *PAOD* Peripheral arterial obstructive disease, *CVD* Cerebral vascular disease, *SD* Senile dementia, *COPD* Chronic obstructive pulmonary disease, *CTD* Connective tissue disease, *GU* Gastroduodenal ulcer, *DM* Diabetes mellitus, *CKD* Chronic kidney disease, *HP* Hemiplegic paralysis, *CL* Chronic leukemia, *ML* malignant lymphadenoma, *LD* Liver disease

### Muscle area and SMI in different subgroups

The muscle area of the T12 pedicle was significantly larger in males than in females (*P* < 0.05), and there was a significant difference in SMI (*P* < 0.05) between males and females. The muscle area and SMI of patients older than 80 years were lower than those of patients under 80, and this difference was statistically significant (*P* < 0.05). There was no significant difference in muscle area or SMI between the long-term hospitalized and short-term hospitalized patients (*P* > 0.05). Similarly, there were no significant differences in muscle area and SMI between the smokers and non-smokers (*P* > 0.05; Table 3).

**Table 3:** Comparison of muscle area and SMI in different subgroups

	Muscle area (cm^2^)	SMI (cm^2^/m^2^)
Gender(n)		
Male(99)	92.92 ± 22.43	33.13 ± 7.74
Female(69)	73.45 ± 11.05	29.73 ± 3.87
*P*-value	< 0.001	< 0.001
Age(n)		
> =80 (113)	80.69 ± 17.71	29.88 ± 5.99
< 80 (55)	93.62 ± 24.34	35.54 ± 6.31
*P*-value	< 0.001	< 0.001
Duration of stay(n)		
> =30 days(7)	82.44 ± 11.55	29.09 ± 5.00
< 30 days(161)	85.03 ± 21.28	31.84 ± 6.69
*P*-value	0.75	0.284
Smoking(n)		
Yes(18)	92.68 ± 24.66	33.85 ± 8.64
No(150)	83.99 ± 20.35	31.48 ± 6.35
*P*-value	0.096	0.153

Muscle area and *SMI* (mean ± SD)

### Index differences between the low muscle mass group and normal muscle mass group

From the SMI values, 121 (72.02%) patients had low muscle mass, including 78 men and 43 women. There were significant differences in the CCI, duration of stay, and serum albumin and hemoglobin levels between the two groups (*P* < 0.05), but no significant differences in BMI (*P* > 0.05; Table 4). There was no obvious correlation with sex.

**Table 4:** Comparison of indexes between low muscle mass group and normal muscle mass group

	low muscle mass (*n* = 121)			normal muscle mass (*n* = 47)		
	All	Male(78)	Female(43)	All	Male(21)	Female(26)
CCI	7.38 ± 1.98^*^	7.50 ± 1.57^Δ^	7.16 ± 2.56^☆^	5.13 ± 0.77^*^	5.33 ± 0.80^Δ^	4.96 ± 0.72^☆^
BMI (kg/m2)	24.62 ± 3.23	24.22 ± 3.28	24.86 ± 2.85	24.27 ± 3.10	24.90 ± 3.84	24.55 ± 2.93
Duration of stay(d)	15.55 ± 12.33^*^	16.94 ± 14.47^Δ^	13.81 ± 6.23^☆^	10.70 ± 5.58^*^	10.19 ± 4.55^Δ^	9.81 ± 5.93^☆^
Alb(g/L)	32.64 ± 3.41^*^	32.28 ± 3.75^Δ^	33.28 ± 2.58^☆^	38.60 ± 2.06^*^	37.97 ± 2.82^Δ^	39.10 ± 0.91^☆^
Hb(g/L)	108.55 ± 13.31^*^	108.68 ± 14.70^Δ^	108.30 ± 10.48^☆^	132.89 ± 10.80^*^	131.05 ± 13.94^Δ^	134.38 ± 7.33^☆^

According to thoracic *SMI* level, the subjects were divided into two groups: low muscle mass group and normal muscle mass group. There were significant differences in *CCI*, duration of stay, Alb and Hb between the two groups (^*^P < 0.05, ^Δ^*P* < 0.05, ^☆^*P* < 0.05). There was no significant difference between the sexes (*P* > 0.05). Alb: albumin, Hb: hemoglobin

### The relationship between CCI and SMI/gait speed

**Fig. 3:**
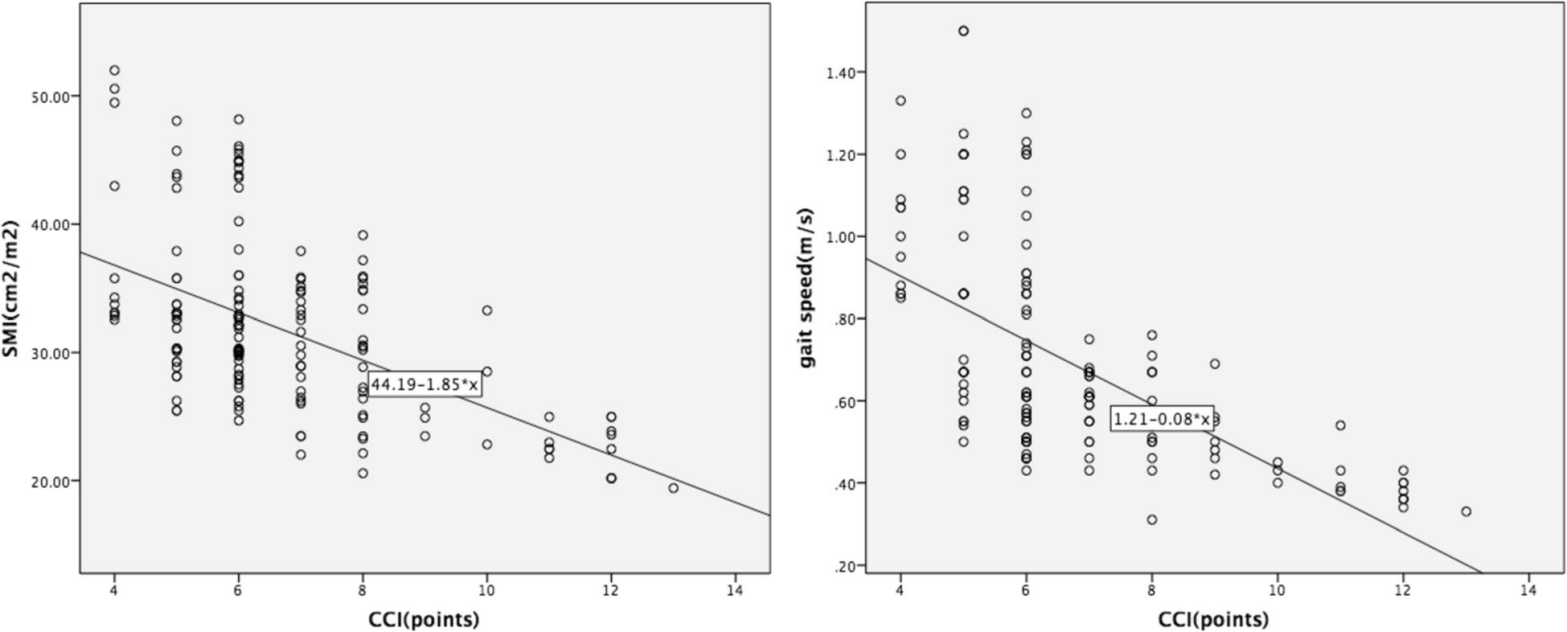
Scatter plot analysis of T12 SMI/gait speed and CCI. The linear correlation method was used to evaluate the association between SMI/gait speed and CCI. SMI: skeletal muscle index, CCI: Charlson comorbidity index

**Fig. 4:**
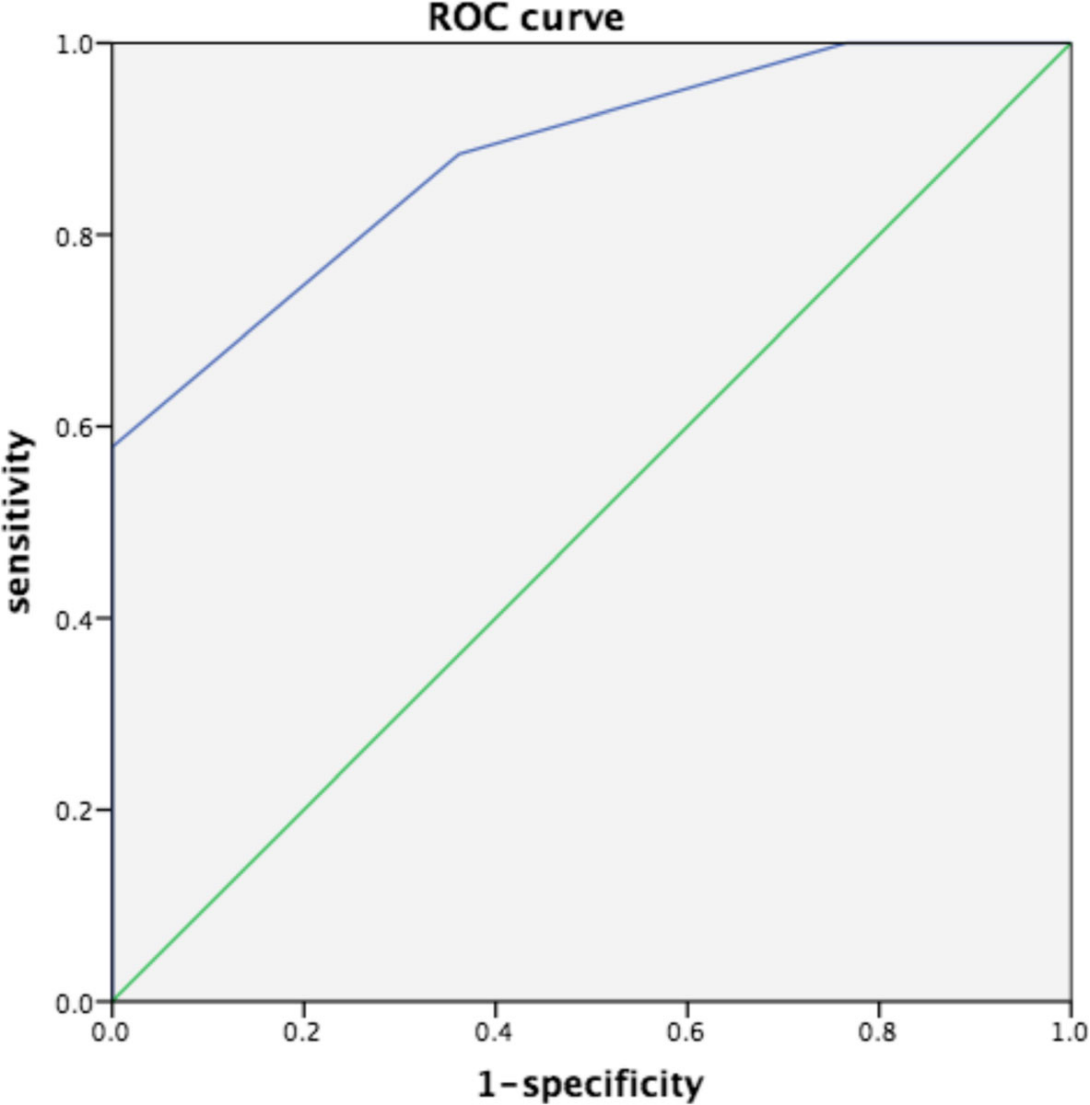
ROC curve of low skeletal muscle mass and CCI. ROC curves were constructed to evaluate the sensitivity and 1-specificity of CCI in the diagnosis of low skeletal muscle mass. ROC: receiver operating characteristic, CCI: Charlson comorbidity index

Linear correlation was used to evaluate the association between CCI and SMI (r = − 0.549, *P* < 0.05), and between CCI and gait speed (r = − 0.614, *P* < 0.05) (Fig. 3). We diagnosed low skeletal muscle mass using the CCI by plotting the receiver operating characteristic curves [12]. The area under the curve (AUC) was 0.879 (*P* < 0.05), the maximum Youden index was 0.579, and its corresponding critical value was 6.5 (Fig. 4).

## Discussion

The global population is aging and the association between age and sarcopenia in older persons is well-known. The prevalence of cardiovascular and cerebrovascular diseases, senile dementia, diabetes mellitus, malignant tumors, and other chronic diseases increases with age [22]. Sarcopenia commonly accompanies aging and numerous diseases. The CCI as the most commonly used tool for comorbidity assessment [23] and can be used to predict the occurrence of sarcopenia, which may be clinically occult. The study of comorbidity in the elderly can include evaluation of mortality, disability, rehospitalization, length of hospital stay, and post-traumatic mortality, which are used to calculate the CCI scores [24]. Diagnostic methods include gait speed, dual-energy X-ray, ultrasound, magnetic resonance imaging, and CT [3]. Physical function is evaluated using gait speed, and CT can accurately distinguish among bone, muscle, fat, and other soft tissues. CT-based SMI scoring is one of the most accurate methods for the diagnosis of sarcopenia [1, 25]. This method has been used for muscle measurement in patients after liver transplantation [26] and in those with pancreatic adenocarcinoma [27]. This measurement of bilateral muscles helps to explain muscle asymmetry associated with scoliosis [28]. To our knowledge, no prior studies have used the CCI to evaluate the risk of sarcopenia. The purpose of this study was to investigate the association between comorbidity and skeletal muscle mass/physical performance by analyzing CCI scores in older people and to determine the risk of sarcopenia from CCI scores.

Our study showed that there were no significant differences in age, duration of stay or BMI between male and female patients. More males than females were smokers. The most recent research [29] has shown that malnourished older people have lower levels of myoglobin. That is, malnourished patients are more likely to have sarcopenia. According to the SMI diagnostic criteria of sarcopenia proposed by Harvard Medical School, the subjects were divided into two groups: a normal muscle mass group and a low muscle mass group. There were differences in the CCI score, duration of stay, serum albumin, and hemoglobin between the two groups. The differences in the above indicators were not significantly related to sex. There was no significant difference in BMI, possibly because BMI is not only related to muscle area and height, but also to muscle, fat and other factors [30]. Through linear correlation analysis, we found a significant negative correlation between SMI/gait speed and the CCI. We analyzed the correlation between the CCI score and age as well as the number and severity of chronic diseases. When multiple diseases coexist, the higher the consumption of nutrients, especially by muscle, the higher the CCI score and the lower the muscle area of older people. In addition, through analysis of the ROC curve and Youden index, we determined that when the CCI score exceeded 6.5, clinicians should be vigilant about determining whether the patient has low skeletal muscle mass. Our results may guide clinical work to assess the risk of sarcopenia in the elderly by using CCI comorbidity assessment tools.

According to the EWGSOP2 update to the original definition of sarcopenia in 2019 [3], skeletal muscle strength is an important component of diagnostic criteria in sarcopenia. A limitation of our study is the lack of skeletal muscle strength data. This may have led to bias in the diagnosis of sarcopenia. We will address this limitation in our next research. Moreover, the inclusion of only patients with chest CT data available may have led to selection bias and may not be representative of the entire elderly population. CT and the use of a stopwatch is a simple and valid technique that can be used to assess treatment effects, but there are subjective factors involved in investigators’ measurements of muscle area and time. Therefore, future studies will need to find objective measurement tools.

In conclusion, in this population, we identified a strong association between the CCI score and SMI/gait speed. We concluded that skeletal muscle mass and physical performance could be assessed using the CCI score. Given the increasing incidence of sarcopenia in the older population, efforts are required to identify new risk factors to predict and prevent the occurrence of sarcopenia.

## Conclusions

We identified an independent association between comorbidity and skeletal muscle mass/physical performance by researching the correlation between the CCI score and SMI/gait speed in individuals aged 65 years and older. Our results suggested an important role of the CCI score in evaluating skeletal muscle mass and physical performance, which may provide important evidence for clinicians to diagnose sarcopenia.

## Data Availability

On reasonable demand, the corresponding author will supply the datasets used and/or analyzed during the current study.

## Abbreviations

AUC: Area under the curve
BMI: Body Mass Index
CCI: Charlson Comorbidity Index
CHD: Chronic heart failure
CKD: Chronic kidney disease
CL: Chronic leukemia
COPD: Chronic obstructive pulmonary disease
CT: Computed tomography
CTD: Connective tissue disease
CVD: Cerebral vascular disease
DM: Diabetes mellitus
GU: Gastroduodenal ulcer
HP: Hemiplegic paralysis
LD: Liver disease
MI: Miocardial infarction
ML: Malignant lymphadenoma
MRI: Magnetic resonance imaging
PAOD: Peripheral arterial obstructive disease
SMI: Skeletal muscle index; SD: Senile dementia

## References

[1] Cruz-Jentoft AJ, Baeyens JP, Bauer JM, Boirie Y, Cederholm T, Landi F, Martin FC, Michel JP, Rolland Y, Schneider SM (2010). Sarcopenia: European consensus on definition and diagnosis: report of the European working group on sarcopenia in older people. Age Ageing.

[2] Cruz-Jentoft A, Bahat G, Bauer J, Boirie Y, Bruyère O, Cederholm T, Cooper C, Landi F, Rolland Y, Sayer A (2019). Sarcopenia: revised European consensus on definition and diagnosis. Age Ageing.

[3] Akishita M, Kozaki K, Iijima K, Tanaka T, Shibasaki K, Ogawa S, Arai H (2018). Chapter 1 definitions and diagnosis of sarcopenia. Geriatr Gerontol Int.

[4] Morley JE, Anker SD, von Haehling S (2014). Prevalence, incidence, and clinical impact of sarcopenia: facts, numbers, and epidemiology-update 2014. J Cachexia Sarcopenia Muscle.

[5] Montero-Fernández N, Serra-Rexach J (2013). Role of exercise on sarcopenia in the elderly. Eur J Phys Rehabil Med.

[6] Liao Y, Peng Z, Chen L, Zhang Y, Cheng Q, Nüssler A, Bao W, Liu L, Yang W (2019). Prospective views for whey protein and/or resistance training against age-related sarcopenia. Aging Dis.

[7] Zemedikun D, Gray L, Khunti K, Davies M, Dhalwani N (2018). Patterns of multimorbidity in middle-aged and older adults: an analysis of the UK biobank data. Mayo Clin Proc.

[8] Roig M, Eng JJ, MacIntyre DL, Road JD, FitzGerald JM, Burns J, Reid WD (2011). Falls in people with chronic obstructive pulmonary disease: an observational cohort study. Respir Med.

[9] Tsur A, Segal Z (2010). Falls in stroke patients: risk factors and risk management. Isr Med Assoc J : IMAJ.

[10] Mak MK, Pang MY (2010). Parkinsonian single fallers versus recurrent fallers: different fall characteristics and clinical features. J Neurol.

[11] McAdams-DeMarco MA, Suresh S, Law A, Salter ML, Gimenez LF, Jaar BG, Walston JD, Segev DL (2013). Frailty and falls among adult patients undergoing chronic hemodialysis: a prospective cohort study. BMC Nephrol.

[12] Bandeen-Roche K, Seplaki C, Huang J, Buta B, Kalyani R, Varadhan R, Xue Q, Walston J, Kasper J (2015). Frailty in older adults: a nationally representative profile in the United States. J Gerontol A Biol Sci Med Sci.

[13] Al-Momani M, Al-Momani F, Alghadir A, Alharethy S, Gabr S (2016). Factors related to gait and balance deficits in older adults. Clin Interv Aging.

[14] Durham-Lee J, Wu Y, Mokkapati V, Paulucci-Holthauzen A, Nesic O (2012). Induction of angiopoietin-2 after spinal cord injury. Neuroscience.

[15] Maeda K, Akagi J (2017). Cognitive impairment is independently associated with definitive and possible sarcopenia in hospitalized older adults: the prevalence and impact of comorbidities. Geriatr Gerontol Int.

[16] Echeverria P, Bonjoch A, Puig J, Estany C, Ornelas A, Clotet B, Negredo E (2018). High prevalence of sarcopenia in HIV-infected individuals. Biomed Res Int.

[17] Wang T, Feng X, Zhou J, Gong H, Xia S, Wei Q, Hu X, Tao R, Li L, Qian F (2016). Type 2 diabetes mellitus is associated with increased risks of sarcopenia and pre-sarcopenia in Chinese elderly. Sci Rep.

[18] Liang-Kung C, Li-Kuo L, Jean W, Prasert A, Tung-Wai A, Kamaruzzaman Shahrul B, Ming-Yueh C, Liang-Yu C, Pi-Shan H, Orapitchaya K (2014). Sarcopenia in Asia: consensus report of the Asian working Group for Sarcopenia. J Am Med Dir Assoc.

[19] Nemec U, Heidinger B, Sokas C, Chu L, Eisenberg R (2017). Diagnosing sarcopenia on thoracic computed tomography: quantitative assessment of skeletal muscle mass in patients undergoing Transcatheter aortic valve replacement. Acad Radiol.

[20] Chang C, Wu J, Mhuircheartaigh J, Hochman M, Rodriguez E, Appleton P, Mcmahon C (2018). Effect of sarcopenia on clinical and surgical outcome in elderly patients with proximal femur fractures. Skelet Radiol.

[21] Ondeck NT, Bovonratwet P, Ibe IK, Bohl DD, McLynn RP, Cui JJ, Baumgaertner MR, Grauer JN (2018). Discriminative ability for adverse outcomes after surgical Management of hip Fractures: a comparison of the Charlson comorbidity index, Elixhauser comorbidity measure, and modified frailty index. J Orthop Trauma.

[22] Clynes MA, Edwards MH, Buehring B, Dennison EM, Binkley N, Cooper C (2015). Definitions of sarcopenia: associations with previous falls and fracture in a population sample. Calcif Tissue Int.

[23] Brilleman SL, Salisbury C (2013). Comparing measures of multimorbidity to predict outcomes in primary care: a cross sectional study. Fam Pract.

[24] Zekry D, Loures Valle BH, Graf C, Michel JP, Gold G, Krause KH, Herrmann FR (2012). Prospective comparison of 6 comorbidity indices as predictors of 1-year post-hospital discharge institutionalization, readmission, and mortality in elderly individuals. J Am Med Dir Assoc.

[25] Chen LK, Liu LK, Woo J, Assantachai P, Auyeung TW, Bahyah KS, Chou MY, Chen LY, Hsu PS, Krairit O (2014). Sarcopenia in Asia: consensus report of the Asian working Group for Sarcopenia. J Am Med Dir Assoc.

[26] Lee C, Cron D, Terjimanian M, Canvasser L, Mazurek A, Vonfoerster E, Tishberg L, Underwood P, Chang E, Wang S (2014). Dorsal muscle group area and surgical outcomes in liver transplantation. Clin Transpl.

[27] Joglekar S, Asghar A, Mott S, Johnson B, Button A, Clark E, Mezhir J (2015). Sarcopenia is an independent predictor of complications following pancreatectomy for adenocarcinoma. J Surg Oncol.

[28] Larson K, Hamlin R, Sprung J, Schroeder D, Weingarten T (2014). Associations between Charlson comorbidity index and surgical risk severity and the surgical outcomes in advanced-age patients. Am Surg.

[29] Sousa AS, Guerra RS, Fonseca I, Pichel F, Amaral TF (2016). Sarcopenia and length of hospital stay. Eur J Clin Nutr.

[30] Fukuoka Yuki, Narita Takuma, Fujita Hiroki, Morii Tsukasa, Sato Takehiro, Sassa Mariko Harada, Yamada Yuichiro (2018). Importance of physical evaluation using skeletal muscle mass index and body fat percentage to prevent sarcopenia in elderly Japanese diabetes patients. Journal of Diabetes Investigation.

